# A novel nucleoside rescue metabolic pathway may be responsible for therapeutic effect of orally administered cordycepin

**DOI:** 10.1038/s41598-019-52254-x

**Published:** 2019-10-31

**Authors:** Jong Bong Lee, Masar Radhi, Elena Cipolla, Raj D. Gandhi, Sarir Sarmad, Atheer Zgair, Tae Hwan Kim, Wanshan Feng, Chaolong Qin, Cecilia Adrower, Catherine A. Ortori, David A. Barrett, Leonid Kagan, Peter M. Fischer, Cornelia H. de Moor, Pavel Gershkovich

**Affiliations:** 1 School of Pharmacy, University of Nottingham, Nottingham, NG7 2RD UK; 2 Department of Pharmaceutics, Ernest Mario School of Pharmacy, Rutgers, The State University of New Jersey, Piscataway, NJ 08854 USA; 3 School of Pharmacy, Universita di Roma Tor Vergata, Rome, 00173 Italy; 4 College of Pharmacy, University of Anbar, Anbar, 31001 Iraq; 5 College of Pharmacy, Catholic University of Daegu, Gyeongsan, 38430 Republic of Korea

**Keywords:** Pharmacology, Drug development

## Abstract

Although adenosine and its analogues have been assessed in the past as potential drug candidates due to the important role of adenosine in physiology, only little is known about their absorption following oral administration. In this work, we have studied the oral absorption and disposition pathways of cordycepin, an adenosine analogue. *In vitro* biopharmaceutical properties and *in vivo* oral absorption and disposition of cordycepin were assessed in rats. Despite the fact that numerous studies showed efficacy following oral dosing of cordycepin, we found that intact cordycepin was not absorbed following oral administration to rats. However, 3′-deoxyinosine, a metabolite of cordycepin previously considered to be inactive, was absorbed into the systemic blood circulation. Further investigation was performed to study the conversion of 3′-deoxyinosine to cordycepin 5′-triphosphate *in vitro* using macrophage-like RAW264.7 cells. It demonstrated that cordycepin 5′-triphosphate, the active metabolite of cordycepin, can be formed not only from cordycepin, but also from 3′-deoxyinosine. The novel nucleoside rescue metabolic pathway proposed in this study could be responsible for therapeutic effects of adenosine and other analogues of adenosine following oral administration. These findings may have importance in understanding the physiology and pathophysiology associated with adenosine, as well as drug discovery and development utilising adenosine analogues.

## Introduction

Adenosine is an endogenous purine nucleoside consisting of adenine and ribose and can be phosphorylated into mono-, di- and triphosphate forms. Adenosine and its various forms play fundamental roles in numerous physiological and pathophysiological processes, ranging from energy transfer, cell signalling to components of nucleic acid and coenzymes^1^. Adenosine itself can induce a wide range of physiological responses of vasodilation, reduction in heart rate, regulation of sympathetic nervous system and antithrombotic effects^2^. Receptors for adenosine are ubiquitously expressed throughout the body and their subtypes (A_1_, A_2A_, A_2B_ and A_3_) possess distinct functions in various systems^3^.

Moreover, the active metabolite of adenosine, adenosine triphosphate (ATP), is a phosphorylated form of adenosine which has well-established important biological roles via both intracellular and extracellular mechanisms^4^. Intracellularly, it is not only associated with cellular metabolism but also with RNA synthesis as a purine nucleoside and second messenger system as part of cAMP^5^. ATP-sensitive potassium ion channels are distributed in the cardiac-skeletal and vascular smooth muscles, which then affect the neurotransmission and contraction of the cells^6^. Intracellular concentration of ATP could also determine whether the behaviour of the cells to chemotherapeutic treatment would be apoptotic or necrotic^7^. For chemotherapy, multidrug resistance is a significant issue leading to reduced efficacy and it is known that ATP-dependent transporters play key role in the resistance mechanism^8^. Such variety of biological and pharmacological effects of ATP has led to efforts in development of adenosine analogues and their phosphorylated forms as therapeutic agents^5,9,10^.

However, little is known about the absorption pathways of adenosine and its analogues following oral administration. In general, it is known that only minimal absorption of adenosine (or its analogues with the adenine group) can be achieved due to extensive metabolism during the absorption process in the gastrointestinal tract^11–13^. It has been reported that rapid metabolism (deamination) of the adenine group prevents absorption in its intact form and this observation has led to the design of analogues that can avoid this route of degradation^14–17^.

Cordycepin (3′-deoxyadenosine) is an adenosine analogue which has been studied extensively from mid-20^th^ century since its isolation and identification as the main active component of the fungus, *Cordyceps militaris*^18^. Cordycepin has been reported to have a diverse range of pharmacological effects of which the most well-studied areas include anti-inflammation, anti-proliferation and pro-apoptosis^19–21^. The wide range of potential therapeutic applications of cordycepin has drawn substantial research interest in the compound which resulted in numerous preclinical *in vivo* efficacy studies in different species^22^. Reports include claims of efficacy in animal models of bone loss^23^, vascular disorder^24^, cognitive dysfunction^25^, sleep disorders^26^, cancer^21,27^, neuronal degeneration^28^, hyperlipidaemia^29^, oxidative stress^30^, hyperglycaemia^31^, asthma and lung inflammation^32^ and viral infection^33^. Indeed, we have recently shown that oral cordycepin is effective in rodent models of osteoarthritis and demonstrated that it acts as an anti-inflammatory pain killer^34^. However, there seems to be lack of data on absorption and disposition of cordycepin as only extremely limited information can be found in the literature^35,36^.

As is the case for adenosine and its analogues, oral absorption and bioavailability of cordycepin is not well understood, despite multiple *in vivo* pharmacology studies performed with oral administration and the oral route being the intended route of administration for cordycepin^23–25,27,29,34,37,38^. The information on the oral absorption processes and the biotransformation of cordycepin is critical in elucidating the exposure-effect relationship, especially given that there is strong evidence that cordycepin needs to be converted to cordycepin 5′-triphosphate (CordyTP) in order to exert at least some of its pharmacological effects^19,39–41^.

In this study, we aimed to elucidate absorption and disposition properties of cordycepin, with potential wider implications for adenosine and its other analogues. The most important finding of this work was that only 3′-deoxyinosine, previously considered an inactive metabolite of cordycepin^42,43^, was absorbed and cordycepin was not found in the blood circulation undetectable (detection limit 2 ng/mL) following oral administration. We also showed that 3′-deoxyinosine can be converted to CordyTP and has anti-inflammatory effects in tissue culture cells. It is likely that similar phenomena also occur with adenosine and its analogues, which would have important implications for understanding of their basic biology, as well as understanding of the biotransformation pathways.

## Materials and Methods

### Materials

Cordycepin (3′-deoxyadenosine, CAS: 73-03-0) was obtained from Carbosynth Ltd (Berkshire, UK). Cordycepin 5′-triphosphate (CordyTP, CAS: 71997-32-5) and 3′-deoxyinosine (CAS: 13146-72-0) were purchased from Santa Cruz Biotechnology (Heidelberg, Germany). Caco-2 cells were purchased from Public Health England (Salisbury, UK). Rat plasma was obtained from Sera Laboratories (West Sussex, UK). Human plasma was purchased from TCS Biosciences (Buckingham, UK). Pentostatin (deoxycoformycin, CAS: 53910-25-1), 2′-deoxyadenosine (CAS: 40627-14-3), 2-chloroadenosine (CAS: 146-77-0), foetal bovine serum (FBS), sodium taurocholate (NaTc), NaCl, NaOH (pellets), NaH_2_PO_4_, KH_2_PO_4_, K_2_HPO_4_, Dulbecco’s Modified Eagle Medium (DMEM), lipopolysaccharide (LPS), S-(4-nitrobenzyl)-6-thioinosine (NBTI), ITu (5-iodotubericidin) and NADPH were obtained from Sigma (Gillingham, UK). All solvents were from Fisher Scientific (Leicestershire, UK) and were of HPLC grade.

### Plasma stability

Stability of cordycepin in plasma was tested using human and rat plasma by following a previously reported method with minor modifications^44^. Cordycepin was spiked into the plasma at 10 µM and the samples were incubated in a shaking incubator (Thermo Scientific MaxQ4000, Waltham, MA, USA) at 37 °C and shaken at 250 rpm. Samples (100 µL) were withdrawn at predetermined time points. Cordycepin is known to be rapidly degraded to 3′-deoxyinosine by the adenosine deaminase enzymes in plasma and pentostatin is often used as an inhibitor of this process^43,45,46^. When pentostatin was tested for stabilisation of cordycepin in plasma, it was spiked 5 min prior to spiking of cordycepin. The experiments were performed in triplicates.

### Plasma protein binding

Human and rat plasma containing 100 nM of pentostatin were used in the study. Cordycepin was spiked into the plasma to give concentrations of 100, 500, 1000 and 5000 ng/mL, and the samples (300 µL) were incubated in the shaking incubator at 250 rpm and 37 °C for 1 h. Following incubation, 100 µL was withdrawn and analysed for *C*_*t*_. The remaining sample was transferred to a filter device (Amicon Ultra-0.5 Centrifugal Filter Units, MWCO 3000, Merck Millipore, Cork, Ireland) and was centrifuged at 14,000 *g* for 10 min at room temperature. Filtrate was collected and analysed for *C*_*f*_. The fraction unbound in plasma (*F*_*ub*_) was calculated by the equation: *C*_*f*_/*C*_*t*_ × 100 = *F*_*ub*_. Non-specific binding of cordycepin to the filter device was tested at the same concentration range using phosphate buffered saline (PBS) instead of plasma. Experiments were conducted in quadruplicates.

### Biorelevant solubility

The three biorelevant fluids of fasted state simulated gastric fluid (FaSSGF, pH 1.6), fasted state simulated intestinal fluid (FaSSIF, pH 6.5) and fed state simulated intestinal fluid (FeSSIF, pH 5.0) were prepared according to previously reported compositions^47^. The test mixtures containing biorelevant fluids and excessive amount of cordycepin were shaken at 250 rpm, 37 °C for 2 h in the shaking incubator. The mixtures were then filtered using Costar Spin-X Centrifuge Tubes (Fisher Scientific, Loughborough, UK) at 2400 *g* for 5 min. The filtrate was subjected to analysis by HPLC-UV. The experiments were conducted in quadruplicates.

### Caco-2 permeability

Caco-2 permeability assays were conducted following previously reported methods with 21-day maintenance on the Corning 24-well Transwell^®^ (Fisher Scientific, Loughborough, UK)^48^. Cordycepin was tested at 200 µM with and without presence of pentostatin (10 µM). Both directions of apical-to-basolateral and basolateral-to-apical were tested. Permeability was assessed for 120 min with sampling every 30 min, and the donor side was sampled after the 120 min to assess remaining concentration of cordycepin (the sensitivity limit of the bioanalytical method did not allow to quantify concentration levels of CordyTP in these samples). The experiments were conducted in triplicates.

### Liver microsomal stability

Human and rat liver microsomes were used in the experiment following previously reported methods^49,50^. Microsomes were used at 0.5 mg protein/mL and cordycepin was tested at 1 µM. Four groups were tested for both species of microsomes: 1) with NADPH (1 mM) and with pentostatin (10 µM); 2) with NADPH and without pentostatin; 3) without NADPH and with pentostatin; and 4) without NADPH and without pentostatin. The reaction mixture was incubated in the shaking incubator at 37 °C for 30 min with sampling every 10 min. The results of intrinsic clearance and the fraction that escapes hepatic metabolism (by both well-stirred and parallel-tube models) were calculated by previously reported equations and physiological parameters^50–52^. The experiments were performed in triplicates.

### *In vitro* experiments using macrophage cells

RAW 264.7 mouse macrophage cells were cultured in DMEM supplemented with 10% FBS. Prior to experiments, cells were washed with PBS and cells were maintained in DMEM with 0.5% FBS or 10% FBS for 24 h. LPS was added at 1 µg/mL, 1 h before compound treatment, when needed. When the cells were analysed for concentration levels of cordycepin, 3′-deoxyinosine and CordyTP, the cells were washed twice with PBS. The cells were then treated with methanol and mixed vigorously before the mixture was harvested. The mixture was filtered using filter tubes mentioned above and the filtrate was analysed by liquid chromatography coupled with tandem mass spectrometry (LC-MS/MS). For determination of relative mRNA levels, RNA was isolated from the lysates using an RNA isolation kit, following manufacturer’s protocol (Promega, Madison, WI, cat no. Z6012). RNA was reverse transcribed to cDNA using SuperScript™ III reverse transcriptase (Invitrogen, Carlsbad, CA). Quantitative polymerase chain reaction (qPCR), was performed with Qiagen Rotor-Gene Q qPCR machine (Qiagen, Hilden, Germany) using Promega SYBR Green GoTaq® qPCR master mix (Promega). Supplementary Information 1 shows the list of qPCR primers used.

### *In vivo* pharmacokinetic experiments

Protocols for the animal experiments in this study were reviewed and approved by the University of Nottingham Ethical Review Committee in accordance with the Animals [Scientific Procedures] Act 1986. Four male Sprague Dawley rats (Charles River Laboratories, UK) of 300–350 g body weight were used. The animals were housed in the Bio Support Unit, University of Nottingham at controlled temperature, 12 h light/dark cycle and free access to food and water. The animals were allowed to acclimatise for at least six days prior to any procedures.

Prior to pharmacokinetic studies, jugular vein cannulation surgery was performed under general anaesthesia (<3% isoflurane inhalation) for blood sampling or intravenous drug administration. Following two nights of recovery from surgery, the animals were intravenously or orally administered with the PBS-based formulation (5.5 mg/mL cordycepin) developed in a previously reported study^22^. The dose was 8 or 20 mg/kg for intravenous administration and 8 or 80 mg/kg for oral administration. Blood samples (approx. 0.2 mL) were collected at predetermined time points into Eppendorf tubes pretreated with pentostatin (10 µM). When whole blood was analysed, an aliquot was withdrawn immediately after mixing with the pretreated pentostatin. Otherwise, the mixture was centrifuged (3000 *g*, 10 min) to obtain plasma and samples were stored within the storage conditions tested prior to analysis.

### Bioanalytical procedures for determination of concentrations

Samples from studies of plasma stability, protein binding, biorelevant solubility and liver microsomal stability were subjected to liquid-liquid extraction and HPLC-UV analysis. To 100 µL of sample, internal standard (2′-deoxyadenosine) was spiked at 10 µg/mL and 300 µL of 0.1 M NaOH was added. Extraction solvent (3 mL) of ethyl acetate-isopropyl alcohol (85:15, v/v) was added and vortex-mixed for 10 min. Samples were then centrifuged at 1160 *g* for 5 min and the supernatant was transferred and dried under N_2_ gas. Dried samples were reconstituted with 100 uL of deionised distilled water (DDW) and 40 µL was injected into the HPLC-UV. The HPLC-UV setup was adopted from a validated method^22^ with addition of a gradient scheme to elute the impurities from samples. Waters Alliance 2695 module with Waters 996 PDA detector was used for the HPLC-UV system with Capcell Pak C18 3 µm, 4.6 × 150 mm as the stationary phase. Mobile phase was a mixture of acetonitrile (A) and DDW (B) at flow rate 0.8 mL/min with a gradient scheme of the following: 0–9 min, B 94%; 9–14 min, B decreased to 20%; 14–24 min, B 20%; 24–26 min, B increased to 94%; and 26–35 min, B 94%. Calibration curves were constructed at a range of 10 – 20000 ng/mL and chromatograms were obtained at 259 nm of wavelength.

Samples from Caco-2 permeability, *in vitro* determination of CordyTP formation and *in vivo* experiments were subjected to protein precipitation followed by LC-MS/MS analysis. To 50 µL of rat plasma sample, 150 µL of internal standard solution (1.67 µg/mL 2-chloroadenosine in methanol) was added. The samples were then vortex-mixed for 10 s and were centrifuged for 3 min at 17949 *g*. Supernatant (20 µL) was collected and diluted 10-fold with DDW before injection into the LC-MS/MS for analysis of cordycepin and 3′-deoxyinosine. For analysis of CordyTP, the supernatant was filtered using filter tubes mentioned above and injected without dilution. Tissue samples were homogenised using Bullet Blender Gold (Next Advance, Inc., Troy, NY) in methanol (1:3 w/v) and centrifuged for 3 min at 17949 *g*. The supernatant was filtered and injected for analysis of CordyTP or diluted 10-fold with DDW for analysis of cordycepin and 3′-deoxyinosine. The LC-MS/MS system was a API4000 QTrap (AB Sciex, Warrington, UK) equipped with HPLC setup of SCL10Avp controller, LC10ADvp pump and SILHTC autosampler (Shimadzu, Milton Keynes, UK). Three different methods were utilised for each compound and multiple reaction monitoring parameters of the MS/MS conditions are shown in Table 1. The HPLC conditions including stationary phases and specific mass spectrometry parameters are shown in Supplementary Information 2. The LC-MS/MS method for cordycepin was fully validated in terms of accuracy, precision, sensitivity, selectivity, linearity and sample storage stability in accordance with the Guidance for Industry, Bioanalytical Method Validation, (2018) published by the US FDA (refer to Supplementary Information 3 for validation procedures and results)^53^.

**Table 1:** Multiple reaction monitoring parameters of the LC-MS/MS conditions of cordycepin, 3′-deoxyinosine and CordyTP.

Compound	ESI mode	Q1 (*m/z*)	Q3 (*m/z*)	DP (V)	EP (V)	CE (eV)	CXP (eV)
**Cordycepin**	+	252.2	136.0	41	10	23	10
3′**-Deoxyinosine**	+	253.2	137.1	66	10	27	10
**CordyTP**	−	490.0	158.8	−100	−10	−42	−7
**2-chloroadenosine**							
**(internal standard)**	+	302.1	170.1	61	10	27	12

ESI, electrospray ionisation; DP, declustering potential; EP, entrance potential; CE, collision energy; CXP, collision cell exit potential.

### Data analysis

Data are presented as mean ± standard deviation (SD). A two-tailed unpaired *t*-test was applied for assessment of statistical significance of differences between two groups. For three or more groups, the statistical significance was assessed by application of a one-way ANOVA followed by Tukey’s multiple comparisons test. A *p*-value lower than 0.05 was deemed statistically significant. Statistical analyses were conducted using GraphPad Prism version 7.01 (GraphPad Software, Inc., La Jolla, CA, USA). Pharmacokinetic parameters were obtained by non-compartmental analysis using Phoenix WinNonlin 6.3 software (Pharsight, Mountain View, CA, USA).

## Results

### *In vitro* biopharmaceutical assessment of cordycepin

**Figure 1:**
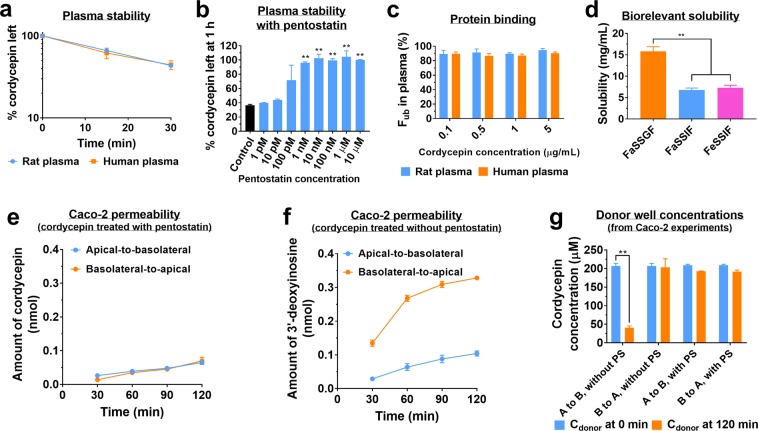
*In vitro* biopharmaceutical assessment of cordycepin (mean ± SD). (**a**) Time-dependent degradation of cordycepin in rat and human plasma (n = 4). (**b**) Stabilisation effect of pentostatin on cordycepin tested in rat plasma at different concentrations of pentostatin (1 pM – 10 µM) (n = 4). **p < 0.01 compared to control group (without pentostatin). (**c**) Plasma protein binding results of cordycepin in rat and human plasma presented as fraction unbound (*F*_*ub*_) in plasma (n = 4). (**d**) Biorelevant solubility of cordycepin tested in fasted state simulated gastric fluid (FaSSGF), fasted state simulated intestinal fluid (FaSSIF) and fed state simulated intestinal fluid (FeSSIF) (n = 4). **p < 0.01. (**e**) Permeability of cordycepin tested using Caco-2 cells with co-administration of pentostatin at both apical-to-basolateral (A to B) and basolateral-to-apical (B to A) directions (n = 3). **(f)** Permeation of 3′-deoxyinosine when Caco-2 cells are treated with cordycepin without co-administration of pentostatin. In this case, permeation of cordycepin was not detected (n = 3). (**g**) Cordycepin concentrations analysed before and after the permeability experiment in the donor chambers (n = 3). **p < 0.01.

The stability of cordycepin was tested in rat and human plasma (Fig. 1a), which showed comparable *ex vivo* stability half-lives (*t*_*1/2*_) between the two species (rat: *t*_*1/2*_ = 25.0 ± 0.7 min; human: *t*_*1/2*_ = 25.8 ± 4.0 min). The inhibition of this metabolism by pentostatin was tested at a wide range of concentrations using rat plasma during 1 h incubation (Fig. 1b). Pentostatin was shown to be a potent inhibitor of the enzyme, exhibiting stabilising effect of cordycepin from at 1 nM. This set of results served as the basis for using pentostatin as stabiliser of cordycepin in the samples during or following *in vitro* and *in vivo* experiments.

Binding of cordycepin (stabilised by pentostatin) to plasma proteins was tested *in vitro* in rat and human plasma using the ultrafiltration method (Fig. 1c). The fraction unbound (*F*_*ub*_) in plasma was > 80% and did not differ across the concentration range tested and between species. Such high *F*_*ub*_ could be expected considering the low lipophilicity of cordycepin (log P = −0.41, ACD/Labs, Toronto, Canada). The non-specific binding of cordycepin to the ultrafiltration tubes was tested with PBS and was found to be negligible (2–3%), which is consistent with tests using other filtration devices^22^.

Solubility of cordycepin was tested in the biorelevant media of FaSSGF, FaSSIF and FeSSIF (Fig. 1d). The solubilities in the three biorelevant fluids were higher than previously reported solubility in water (5.0 ± 0.1 mg/mL)^22^. Since nucleoside analogues, including cordycepin, are weak bases, the solubility was significantly higher in FaSSGF compared to the two simulated intestinal fluids. Solubility in FaSSIF and FeSSIF was comparable, indicating that food effect on oral absorption of cordycepin would be minimal.

Permeability was tested *in vitro* using Caco-2 cells to predict intestinal absorption of cordycepin (Fig. 1e–g). Caco-2 cells mimic the gastrointestinal environment by forming villi-like shapes and expressing brush border enzymes^54,55^. Time-dependent permeation of cordycepin itself (Fig. 1e) could only be obtained in the presence of pentostatin. When tested without pentostatin, cordycepin permeation was not detected in either apical-to-basolateral or basolateral-to-apical directions suggesting a rapid metabolism under the tested conditions. Accordingly, only the permeation of 3′-deoxyinosine, the metabolite of cordycepin, was detected when cordycepin was tested in the absence of pentostatin (Fig. 1f). Our results (Fig. 1e–f) suggest that cordycepin was most probably metabolised by adenosine deaminase enzyme secreted by Caco-2 cells during the permeation. This was further confirmed by analysis of donor chambers for remaining concentrations of cordycepin at the end of the permeability experiments (at 120 min). We found that cordycepin was significantly degraded when it was applied to the apical side without pentostatin (Fig. 1g). When cordycepin was applied with pentostatin to the apical side or without pentostatin to the basolateral side, the concentration of cordycepin remained stable. This is most likely because Caco-2 cells are oriented with villi and enzymes secretion to the apical side and therefore the metabolising enzymes are expected to be present in the apical side only. These data suggest that cordycepin will likely be completely deaminated before it has a chance to be absorbed in the intestine. The bioanalytical method used in this study was not able to quantify levels of CordyTP in these samples. This could be because of two plausible reasons: 1) CordyTP produced by the Caco-2 cells could have been rapidly metabolised by ecto-nucleotidase enzymes upon release into the extracellular environment, which would also apply for plasma samples^56^; 2) Caco-2 cells may not be able to produce CordyTP at all.

Rat and human liver microsomes were used to test the microsomal stability of cordycepin for prediction of hepatic metabolism (Table 2). Intrinsic clearance (*CL*_*int*_) was calculated for the rate of hepatic metabolism and the fraction that escapes hepatic metabolism (*F*_*h*_) was obtained from the *CL*_*int*_. The presence of NADPH did not affect the *CL*_*int*_ in both species. It indicates that the CYP-catalysed reactions are not the main contributors in metabolism of cordycepin, since the activity of these enzymes would depend on the presence of the cofactor, NADPH^51^. On the other hand, the *CL*_*int*_ was substantially affected by the presence of pentostatin which demonstrated that the degradation by adenosine deaminase is predominant in hepatic metabolism. The *CL*_*int*_ was significantly higher in rat liver microsome compared with that of human, which is consistent with previous observations for many other compounds^57^. The *F*_*h*_ calculated from *CL*_*int*_ was 10–13% by the well-stirred model and close to 0% by the parallel-tube model^51^. Therefore, it was predicted that bioavailability of cordycepin could potentially be heavily affected by the hepatic first-pass metabolic loss following oral administration.

**Table 2:** Liver microsomal stability of cordycepin tested with rat and human liver microsomes (mean ± SD, n = 3).

	Rat liver microsome				Human liver microsome			
NADPH	+	+	−	−	+	+	−	−
Pentostatin	+	−	+	−	+	−	+	−
CL_int_ (mL/min/kg)	ND	392.2 ± 31.3	ND	390.0 ± 51.4	ND	181.4 ± 13.9	ND	179.1 ± 5.3
F_h_ (%), well-stirred model^a^	ND	12.4 ± 0.9	ND	12.5 ± 1.5	ND	10.3 ± 0.7	ND	10.4 ± 0.3
F_h_ (%), parallel-tube model^a^	ND	0.1 ± 0.0	ND	0.1 ± 0.1	ND	0.0 ± 0.0	ND	0.0 ± 0.0

NADPH, Nicotinamide adenine dinucleotide phosphate; CL_int_, intrinsic clearance; F_h_, fraction that escapes hepatic metabolism; ND, not determined.

The CL_int_ and F_h_ for groups indicated ND could not be calculated as cordycepin was stable for the time period tested.

^a^Well-stirred and parallel-tube models were adopted from ref.^51^

### *In vivo* pharmacokinetic experiments

**Figure 2:**
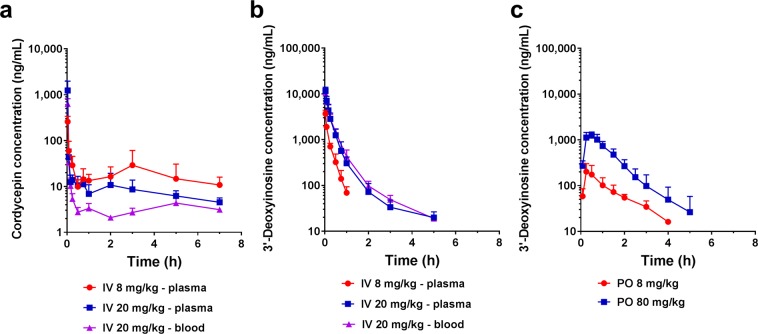
*In vivo* pharmacokinetic profiles of cordycepin and its metabolite, 3′-deoxyinosine, following administration of cordycepin in rats (mean ± SD). (**a**) Profiles of cordycepin following intravenous administration of cordycepin at 8 or 20 mg/kg (n = 4). (**b**) Profiles of 3′-deoxyinosine following intravenous administration of cordycepin at 8 or 20 mg/kg (n = 4). (**c**) Profiles of 3′-deoxyinosine following oral administration of cordycepin at 8 or 80 mg/kg (n = 5).

Cordycepin was administered intravenously (8 and 20 mg/kg) and orally (8 and 80 mg/kg) to rats for assessment of *in vivo* pharmacokinetics of cordycepin and its metabolite, 3′-deoxyinosine (Fig. 2). Intravenous administration profiles of cordycepin showed that cordycepin was rapidly distributed in the system followed by a fluctuating plasma elimination phase (Fig. 2a). Despite such an unusual disposition pattern with fluctuating plasma elimination phase following intravenous bolus administration, a very similar phenomenon has been previously reported for another nucleoside analogue, which suggests that this could be a class nucleoside-type compounds behaviour^58^ (Fig. 2a). Partitioning of cordycepin to erythrocytes or other blood cells was proposed as a possible explanation for the unusual elimination phase and therefore concentration in whole blood was also measured for 20 mg/kg dose group (explained further in Discussion section). However, the blood/plasma ratio turned out to be only 0.68 ± 0.27 and similar unusual disposition pattern was also observed for concentrations of cordycepin in whole blood. Interestingly, the profiles of the metabolite 3′-deoxyinosine following intravenous bolus of cordycepin, exhibited a typical plasma concentration-time profile following intravenous administration (Fig. 2b). Moreover, the profiles of 3′-deoxyinosine in plasma and whole blood following administration of cordycepin (20 mg/kg) were superimposable.

More importantly, when cordycepin was administered to rats orally, it was not detected in plasma at all, at any time point, even with the dose as high as 80 mg/kg. Interestingly however, the metabolite 3′-deoxyinosine was found to be absorbed systemically instead and the plasma concentration-time profiles depicted in Fig. 2c demonstrated high concentrations of 3′-deoxyinosine following oral administration of cordycepin.

Pharmacokinetic parameters obtained for cordycepin and 3′-deoxyinosine following administration of cordycepin are shown in Tables 3 and 4, respectively. The cordycepin concentration extrapolated to time zero (*C*_0_) following intravenous administration was increased 13.8-fold when the dose was increased from 8 to 20 mg/kg (Table 3). Nevertheless, the area under the concentration-time curve (*AUC*) only increased by 1.18-fold. Pharmacokinetics of 3′-deoxyinosine following intravenous bolus also did not show dose-linearity (Table 4). Following intravenous administration, the clearance (*CL*) was found to increase with the dose (between 8 and 20 mg/kg), indicating saturation of the elimination mechanisms. Likewise, the *AUC/Dose* following oral administration showed a decrease when dose was increased from 8 to 80 mg/kg, which suggests a saturable absorption process. Therefore, the bioavailability of 3′-deoxyinosine achievable by oral administration of cordycepin (*F*_*oral*_) was calculated based on the *AUC* values obtained at lower dose level of 8 mg/kg only (36.8 ± 11.3%). Pharmacokinetic profiles and parameters of cordycepin itself following oral administration of cordycepin could not be calculated as no cordycepin was detected after oral administration despite the very sensitive analytical methodology utilised in this work (limit of quantification = 2 ng/mL).

**Table 3:** Pharmacokinetic parameters of cordycepin following intravenous administration of cordycepin.

Route of administration	Intravenous	
Dose (mg/kg)	8	20
C_0_ (ng/mL)	890 ± 576	12298 ± 8844
AUC_0→t_ (ng·h/mL)	196 ± 94	232 ± 106
AUC_0→t_/Dose (ng·h·kg/mL/mg)	25 ± 12	12 ± 5
CL (L/h/kg)	103 ± 52	91 ± 41

C_0_, concentration extrapolated to time zero; AUC_0→t_, area under the concentration-time curve from time zero to the last time point observed; CL, clearance.

**Table 4:** Pharmacokinetic parameters of 3′-deoxyinosine following intravenous and oral administration of cordycepin.

Route of administration	Intravenous		Oral	
Dose (mg/kg)	8	20	8	80
C_0_ or C_max_ (ng/mL)	5729 ± 1633	16347 ± 3871	202 ± 98	1280 ± 267
AUC_inf_ (ng·h/mL)	723 ± 177	2736 ± 720	297 ± 69	1707 ± 555
AUC_inf_/Dose (ng·h·kg/mL/mg)^a^	90 ± 22	137 ± 36	37 ± 9	21 ± 7
T_max_ (h)	—	—	0.25	0.5
t_1/2_ (h)	0.29 ± 0.09	0.80 ± 0.29	1.11 ± 0.39	0.85 ± 0.36
CL (L/h/kg)^b^	11.5 ± 2.7	7.8 ± 2.2	—	—
V_ss_ (L/kg)	2.8 ± 0.3	3.0 ± 0.7	—	—
F_oral_ (%)^c^	—	—	36.8 ± 11.3	—

C_max_, maximum concentration observed, AUC_inf_, area under the concentration-time curve from time zero to infinity; T_max_, time of maximum concentration observed; t_1/2_, elimination half-life; V_ss_, volume of distribution at steady state; F_oral_, oral bioavailability of 3′-deoxyinosine achievable by administration of cordycepin. Refer to Table 3 for abbreviation of other parameters.

^a^Significant difference between AUC_inf_/Dose of 8 and 80 mg/kg oral administration groups was found (p < 0.05).

^b^Significant difference between CL of 8 and 20 mg/kg intravenous administration groups was found (p < 0.05).

### Macrophage derived cells can generate CordyTP from 3′-deoxyinosine

**Figure 3:**
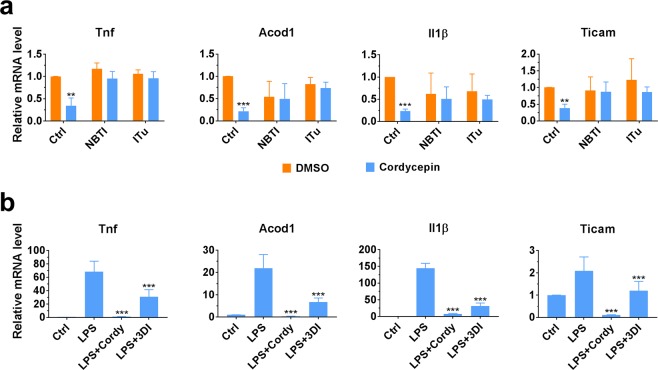
Repression effect on inflammatory gene expression. (**a**) NBTI or ITu treatment prior to cordycepin treatment suppresses effect of cordycepin. RAW264.7 cells were treated with 10 µM NBTI, 100 nM ITu or DMSO (Ctrl, control) 15 min prior to cordycepin treatment. The cells were stimulated with LPS 1 h after cordycepin treatment (mean ± SD, n = 3). **p < 0.01; ***p < 0.001 compared to DMSO treatment (labelled as Ctrl). (**b**) 3′-deoxyinosine exerts same repression effect as cordycepin, although to a lesser degree. RAW264.7 cells were treated with 20 µM cordycepin, 20 µM 3′-deoxyinosine (3DI) or DMSO (Ctrl, control) for 1 h prior to LPS stimulation (mean ± SD, n = 3). **p < 0.01; ***p < 0.001 compared to without compound treatment (labelled as LPS).

It is intriguing that no intact cordycepin was detected in plasma in *in vivo* studies in rats following oral administration, apparently contradicting numerous studies reporting efficacy of cordycepin when given orally, including our own on osteoarthritis pain^23–25,27,29,34,37,38^. Although 3′-deoxyinosine has been previously considered an inactive metabolite, its potential contribution towards efficacy needed further investigation in light of these unexpected pharmacokinetic findings (explained further in Discussion section). Firstly, we sought to confirm that cordycepin needs to be converted intracellularly into CordyTP in order to exert its therapeutic effect. Macrophage-like RAW264.7 cells have been previously reported to be sensitive to cordycepin^59^. The cells were treated with adenosine transporter inhibitor (NBTI), adenosine kinase inhibitor (ITu) or DMSO prior to cordycepin treatment. The results show that NBTI or ITu treatments inhibit the inhibitory effect of cordycepin on expression of inflammatory genes (Fig. 3a). It indicates that indeed cordycepin needs to be transported into the cell and also be phosphorylated intracellularly to have its therapeutic effects which is in agreement with previous reports^19,39–41^. Secondly, it was tested whether 3′-deoxyinosine can manifest the similar therapeutic effects of cordycepin on inflammatory gene expression. RAW264.7 cells were treated with cordycepin or 3′-deoxyinosine prior to stimulation with LPS for inflammatory response. Although 3′-deoxyinosine is not as potent as cordycepin, it also suppressed expression of inflammatory genes (Fig. 3b).

**Figure 4:**
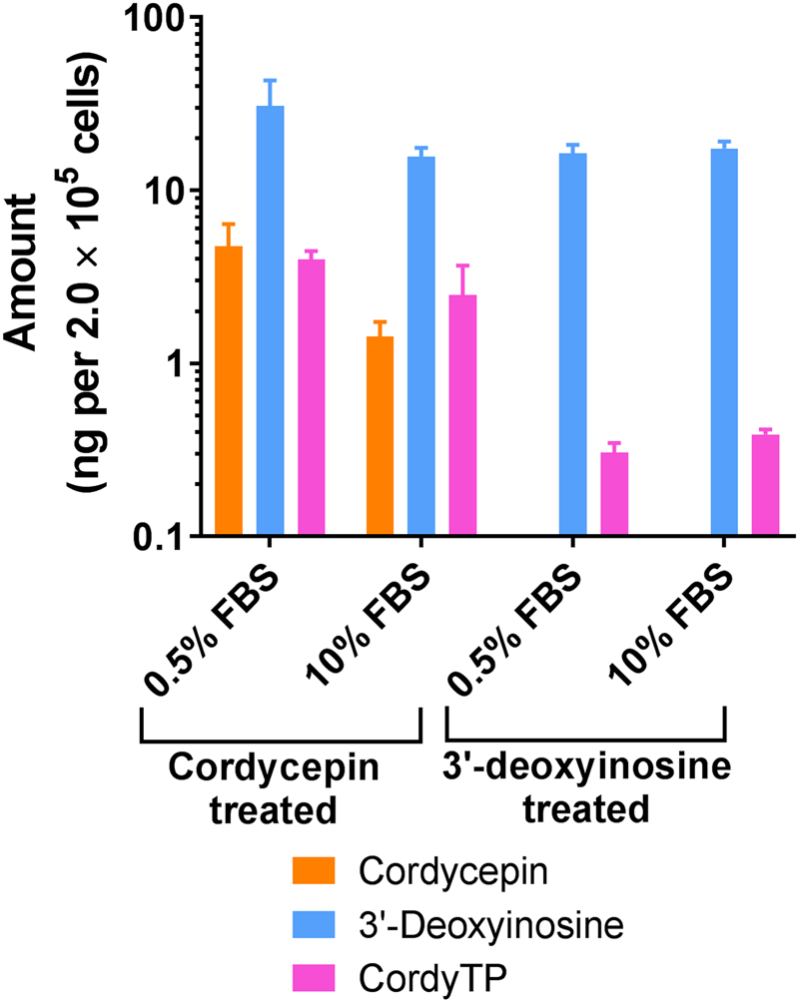
Levels of cordycepin, 3′-deoxyinosine and CordyTP found following *in vitro* treatment with cordycepin or 3′-deoxyinosine. RAW264.7 cells maintained in 0.5 or 10% (foetal bovine serum (FBS) conditions were treated with cordycepin or 3′-deoxyinosine at 20 µM for 6 h (mean ± SD, n = 3).

Our data in Fig. 3a support the idea that CordyTP is the substance responsible for the pharmacological effects related to cordycepin^19,39–41^. Therefore, the possibility of RAW264.7 cells generating CordyTP from 3′-deoxyinosine was tested. We found that the macrophages were indeed able to produce CordyTP from 3′-deoxyinosine (although at an approximately 10-fold lower ratio compared to CordyTP generated from cordycepin) (Fig. 4). These results indicate that 3′-deoxyinosine, a compound which was previously considered an inactive metabolite, could have a major role in therapeutic effects following oral administration of cordycepin.

## Discussion

With the various physiological roles played by adenosine, extensive research has been conducted into development of adenosine analogues as selective and efficient therapeutic agents. However, there has been lack of information on pharmacokinetics of adenosine and its analogues following oral administration. In this study, we aimed to characterise absorption and disposition properties of one of important adenosine analogues, cordycepin, which would propose potential application to adenosine itself and its other analogues. Up to date, only few studies have been conducted on pharmacokinetics of cordycepin^35,36^. Wei *et al*. reported a medicinal chemistry study where prodrugs of cordycepin were synthesised and evaluated following oral administration in mice. However there is minimal information available for the analytical method used and the actual concentrations reported are not all within the calibration curve range, which inevitably would have affected the pharmacokinetic evaluations^35^. Tsai *et al*. conducted pharmacokinetic studies in rats but the information is rather insufficient as unfortunately blood samples were taken sparsely only in the 30–90 min time range following intravenous administration^36^.

In this study, we utilised a fully validated and sensitive bioanalytical method to elucidate the *in vivo* plasma pharmacokinetic profiles of cordycepin. Cordycepin showed rapid disposition following intravenous administration (Fig. 2a). *In vitro* biopharmaceutical assessment showed that cordycepin has low protein binding (Fig. 1) with high hepatic (Table 2) and plasma clearance (Fig. 1), which is consistent with such rapid disposition kinetics *in vivo*. However, the multiple peaks pattern in the elimination phase was unexpected and unusual. Interestingly, this irregular pattern has been previously reported for another adenosine analogue, 3-deazaneplanocin A^58^. Excessive partitioning of cordycepin to blood cells was initially proposed as a possible cause of this phenomenon. However, simultaneous determination of cordycepin concentrations in whole blood and plasma showed that the blood-to-plasma ratio was in fact <1. Moreover, the multiple peaks were also observed in whole blood concentration profiles (Fig. 2a). Enterohepatic recirculation was ruled out as a possibility because no cordycepin was found to be orally absorbed. Therefore, ours as well as previous findings for this unusual disposition profile following intravenous administration of adenosine analogue^58^ do not fully explain this phenomenon. Although this was not the main focus in the current work, this unusual disposition profile warrants further investigation in future studies.

The main finding in this work was that no cordycepin was found in the systemic circulation following oral administration despite relatively high administered dose. Biorelevant solubility results indicated that solubility of cordycepin in the gastrointestinal environment is high and therefore is highly unlikely to be the reason for lack of oral bioavailability (Fig. 1). On the other hand, the apparent permeability coefficient (*P*_*app*_) calculated from Fig. 1a was 0.11 ± 0.01 × 10^−6^ cm/s (A to B), indicating that cordycepin is a compound with poor permeability^48^. It should also be noted that this *P*_*app*_ could only be obtained when pentostatin was used to inhibit enzymatic activity of adenosine deaminase. In the absence of pentostatin, cordycepin was not detected in the receiver compartment probably due to the rapid deamination process. This observation is in line with a previous report in which an increasing amount of inosine was found when adenosine was perfused into the gastrointestinal tract^12^. Low permeability data, taken together with the high hepatic first-pass effect predicted from liver microsomal studies (Table 2), could explain the lack of oral bioavailability of cordycepin. The abovementioned study on 3-deazaneplanocin A tested different routes of administration and they also did not observe any meaningful plasma concentration-time profile following oral administration^58^. However, there are a number of *in vivo* studies reporting substantial efficacy of cordycepin for different indications following oral administration^23–25,27,29,34,37,38^.

In order to understand the apparent contradiction of the lack of oral bioavailability of cordycepin and multiple reports of its therapeutic effects following oral administration, we searched for possible metabolites that could have been potentially absorbed following oral administration of cordycepin. We found that 3′-deoxyinosine appeared at high levels in the systemic circulation *in vivo* following oral administration of cordycepin in rats. As a result, the bioavailability of 3′-deoxyinosine following oral administration of cordycepin was as high as 36.8%. (Fig. 2c). Indeed, the *in vitro* Caco-2 permeability assay also revealed that when cordycepin is applied, 3′-deoxyinosine permeated the cell monolayer instead of cordycepin (Fig. 1f). This *in vitro* experiment suggests that cordycepin is rapidly metabolised during the absorption processes and intact cordycepin could not penetrate the intestinal wall unless it was co-treated with pentostatin. In addition, the *P*_*app*_ of 3′-deoxyinosine was still quite low (approximately 0.2 × 10^−6^ cm/s (A to B)), while the *F*_*oral*_ of 3′-deoxyinosine was 36.8% (Table 4). This indicates possibility of involvement of active transport in the gut that may facilitate absorption of 3′-deoxyinosine.

Although 3′-deoxyinosine is bioavailable, it has been suggested previously that 3′-deoxyinosine is an inactive metabolite, while CordyTP is the active moiety responsible for therapeutic effects of cordycepin^39,42,43^. In this study, we showed that 3′-deoxyinosine suppresses expression of the same genes as cordycepin does, although with lower potency (Fig. 3b). Nevertheless, since 3′-deoxyinosine has been reported previously as an inactive metabolite^42,43^, we hypothesised that some cells may have a metabolic pathway that can convert 3′-deoxyinosine to CordyTP. In order to test this hypothesis, we assessed the ability of a macrophage cell line to generate CordyTP from 3′-deoxyinosine *in vitro*. Although at significantly lower levels compared with cordycepin treatment, the application of 3′-deoxyinosine indeed resulted in detectable levels of CordyTP (Fig. 4). This difference in levels of generated CordyTP is consistent with studies reporting enhanced effect of cordycepin when pentostatin is co-administered^42,43,46^. This is because pentostatin inhibits metabolism of cordycepin and therefore greater amounts of cordycepin can be taken up by the cells to convert it into CordyTP. Further studies will be needed to investigate whether the conversion of 3′-deoxyinosine to CordyTP *in vivo* could produce levels of CordyTP which are high enough to exert the same pharmacodynamic effects.

**Figure 5:**
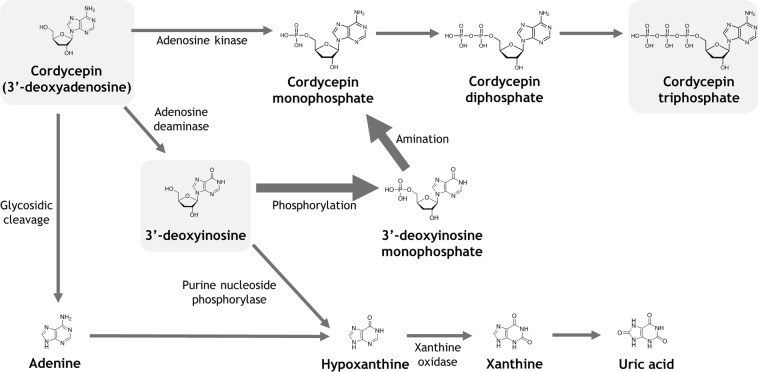
Proposed metabolic pathways of cordycepin. The thicker arrows indicate the novel nucleoside rescue metabolic pathway proposed in this study.

A pathway for conversion of 3′-deoxyinosine into CordyTP which could explain the phenomenon is suggested in Fig. 5. In fact, there have been previous reports of this conversion, although the research was conducted mostly with parasites rather than mammalian cells^60,61^. Nevertheless, the conversion of inosine and its analogues to their respective adenosine triphosphate analogues has been also demonstrated using mammalian cells^62^. A similar pathway had been proposed for another adenosine analogue, 2′,3′-dideoxyadenosine, but the authors have not confirmed the proposed mechanism experimentally^13^. It is important to investigate in the future work which enzymes are responsible for the conversion of 3′-deoxyinosine to CordyTP. If this nucleoside recovery pathway is cell-type specific, the *in vivo* effects of cordycepin observed in multiple previous studies may be due to effects on a limited number of cell types and tissues.

In conclusion, in this work we have demonstrated that despite numerous previous studies showing therapeutic effects of cordycepin following oral administration, it is in fact not orally bioavailable. However, a metabolite previously considered inactive (3′-deoxyinosine) has significant bioavailability following oral administration of cordycepin. Moreover, we have shown that 3′-deoxyinosine could be converted to the active moiety CordyTP in mammalian cells, and therefore could be responsible for therapeutic effects of cordycepin when it is administered orally. These findings provide an important insight into the mechanisms of therapeutic effects of cordycepin. Moreover, this metabolic pathway could play an important role in activity of adenosine and other adenosine analogue drugs. The metabolised products (i.e. the apparently inactive metabolite of cordycepin, 3′-deoxyinosine) are available in the systemic circulation instead and can return to the phosphorylation pathway of their parent form as shown in this study. If drug candidates can be designed with this pathway in mind, extensive derivatisation that focuses on bypassing the metabolism and delivering the intact form could be avoided.

## Supplementary information

Supplementary Info
