# Supplementary material for: A novel nucleoside rescue metabolic pathway may be responsible for therapeutic effect of orally administered cordycepin
Source: Sci Rep. 2019 Oct 31;9:15760. doi: 10.1038/s41598-019-52254-x (PMC6823370; doi:10.1038/s41598-019-52254-x)
Supplement: Supplementary file 1 — Supplementary Info [file 41598_2019_52254_MOESM1_ESM.docx]

**Supplementary Information to:**

**A novel nucleoside rescue metabolic pathway may be responsible for therapeutic effect of orally administered cordycepin**

Jong Bong Lee^1,2^, Masar Radhi^1^, Elena Cipolla^1,3^, Raj D Gandhi^1^, Sarir Sarmad^1^, Atheer Zgair^1,4^, Tae Hwan Kim^5^, Wanshan Feng^1^, Chaolong Qin^1^, Cecilia Adrower^1,3^, Catharine A. Ortori^1^, David A. Barrett^1^, Leonid Kagan^2^, Peter M. Fischer^1^, Cornelia H. de Moor^1^, Pavel Gershkovich^1,^*

^1^ School of Pharmacy, University of Nottingham, Nottingham, UK, NG7 2RD

^2^ Department of Pharmaceutics, Ernest Mario School of Pharmacy, Rutgers, The State University of New Jersey, Piscataway, NJ, USA, 08854

^3^ School of Pharmacy, Universita di Roma Tor Vergata, Rome, Italy, 00173

^4^ College of Pharmacy, University of Anbar, Anbar, Iraq, 31001

^5^ College of Pharmacy, Catholic University of Daegu, Gyeongsan, Republic of Korea, 38430

***Corresponding author:** Pavel Gershkovich, PhD

School of Pharmacy, Centre for Biomolecular Sciences

University of Nottingham, University Park

Nottingham, UK

NG7 2RD

Tel:  +44 (0) 115 846 8014

Fax: +44 (0) 115 951 3412

Email: [pavel.gershkovich@nottingham.ac.uk](mailto:pavel.gershkovich@nottingham.ac.uk)

**Supplementary Information 1.** List of qPCR primers used in the study

| **Gene** | **Sequence of forward primer** | **Sequence of reverse primer** |
| --- | --- | --- |
| Tnf | CTATGGCCCAGACCCTCACA | CCACTTGGTGGTTTGCTACGA |
| Il1β | AGATGAAGGGCTGCTTCCAAA | GGAAGGTCCACGGGAAAGAC |
| Acod1 | ACTCCTGAGCCAGTTACCCT | CTGTGACAGACTTGAGCATCAT |
| Ticam | CTGCTCTGCTCCACAAAACC | AAAAGACAGTGTGGATGCCG |
| Cxcl2 | TGAACAAAGGCAAGGCTAACTG | ACATCAGGTACGATCCAGGC |

**Supplementary Information 2.** HPLC conditions and specific mass spectrometry parameters

The LC-MS/MS system consisted of a HPLC setup (SCL10Avp controller, LC10ADvp pump and SILHTC autosampler) from Shimadzu (Milton Keynes, UK) coupled with API4000 QTrap (AB Sciex, Warrington, UK) for mass spectrometry. The stationary phase for chromatographic separation was a Kinetex C18, 50 × 2.1 mm, 2.6 µm particle size column protected with SecurityGuard 2 × 4 mm (Phenomenex, Macclesfield, UK) and the temperature was maintained at 50 °C. The mobile phase was a mixture of (A) ammonium acetate (5 mM, no pH modification) and (B) acetonitrile with the following gradient scheme: 0-0.5 min, B-5%; 0.5-2.0 min, B increased to 75%; 2.0-3.5 min, B-75%; 3.5-3.7 min, B decreased to 5%. The run time was 5 min with flow rate of 0.35 mL/min. The electrospray ionisation was operated in positive mode with ion source temperature of 350 °C, nebulising gas of 35 psig, turbo gas of 25 psig and entrance potential (EP) of 10 V. The parameters for multiple reaction monitoring were used as listed in Table 1 with dwell time of 200 msec.

**Supplementary Information 3.** Validation procedures and results

Current method was fully validated in accordance with Guidance for Industry, Bioanalytical Method Validation, (2001) published by the US FDA.

1. Accuracy and precision

Accuracy and precision of the bioanalytical method were assessed by preparing and analysing six replicates of lower limit of quantification (LLOQ), LQC, MQC and HQC samples for intra-day (on the same day) and inter-day (different days) validation. Accuracy was expressed as relative error (RE) which was the bias from the nominal concentration. Precision was expressed as relative standard deviation (RSD) representing coefficient of variation. The criteria of RE within ±15% and RSD ≤15% were applied to LQC, MQC and HQC, and RE within ±20% and RSD ≤20% to LLOQ for acceptable values.

2. Sensitivity, selectivity and linearity

The LLOQ was defined as the lowest concentration tested that resulted in acceptable criteria in intra-day and inter-day validation. The analyte response at the LLOQ was also compared with blank response as per the US FDA guidance and ratio of >5 was deemed acceptable [47]. The linearity of calibration curves was assessed by the correlation coefficient (*r^2^*) values in the range of 2 – 50000 ng/mL and *r^2^*>0.99 was considered to be acceptable.

3. Matrix effect

Matrix effect on signal intensity was tested by comparing samples of spiked plasma with samples prepared by diluting the working standard solutions with 10% methanol in DDW. Data was expressed assuming response from working standard solution samples as 100%. It was tested at levels of LQC, MQC and HQC.

4. Stability

Stability of cordycepin was tested in different conditions. Room temperature stability in rat plasma was tested for 4 h with and without 10 µM of pentostatin. Samples withdrawn at pre-determined time points were prepared and analysed for stability and degradation rate of cordycepin in rat plasma.

Sample storage stability studies were conducted with QC samples (LQC, MQC and HQC; 5 replicates for each condition). All samples were spiked into rat plasma containing 10 µM of pentostatin, which was used to stabilise cordycepin. Short-term (benchtop) stability was tested after storing spiked QC samples for 4 h at room temperature. Long-term stability was tested in storage condition of -80 °C for 5 days and 4 weeks. Freeze-thaw cycle stability was tested after undergoing three cycles between -80 °C and room temperature. Autosampler stability was tested by analysing processed QC samples after storage at 4 °C for 24 h. All stability samples were quantified with freshly prepared calibration curve samples. Stock solution stability was tested by analysing five different aliquots of 1000 ng/mL working standard solution stored for 6 h at room temperature and comparing to a fresh working standard solution.

5. Results

**Table 1. Inter-day and intra-day validation for accuracy and precision of the bioanalytical method (mean SD, n = 6)**

| ***Level*** | **Concentration (ng/mL)** | **Intra-day** | |  | **Inter-day** | |
| --- | --- | --- | --- | --- | --- | --- |
|  |  | **Accuracy (RE, %)** | **Precision (RSD, %)** |  | **Accuracy (RE, %)** | **Precision (RSD, %)** |
| **LLOQ** | 2 | 99.9 | 10.3 |  | 100.8 | 6.3 |
| **LQC** | 5 | 100.3 | 8.7 |  | 97.1 | 5.3 |
| **MQC** | 80 | 103.9 | 5.3 |  | 103.2 | 6.4 |
| **HQC** | 4000 | 106.3 | 5.3 |  | 105.3 | 4.7 |

LLOQ, lower limit of quantification; LQC, low quality control; MQC, medium quality control; HQC, high quality control.

**Table 2. Sample stability results at various storage conditions and matrix effect of the bioanalytical method (mean ± SD, n = 5)**

| ***Level*** | **Short-term**  **(4 h, RT)** | **Freeze-thaw**  **(3 cycles)** | **Autosampler**  **(24 h, 4°C)** | **Long-term**  **(5 days, -80°C)** | **Long-term**  **(4 weeks, -80°C)** | **Matrix effect**  **(%)** |
| --- | --- | --- | --- | --- | --- | --- |
| **LQC** | 104.7 ± 9.2 | 104.5 ± 7.4 | 113.8 ± 8.9 | 107.4 ± 3.8 | 93.5 ± 7.0 | 108.5 ± 8.4 |
| **MQC** | 98.2 ± 7.3 | 113.1 ± 13.1 | 91.6 ± 3.6 | 110.0 ± 3.3 | 113.5 ± 3.3 | 98.3 ± 5.4 |
| **HQC** | 107.4 ± 2.9 | 106.7 ± 7.4 | 100.4 ± 3.4 | 98.6 ± 5.4 | 103.8 ± 1.5 | 98.5 ± 3.8 |

LLOQ, lower limit of quantification; LQC, low quality control; MQC, medium quality control; HQC, high quality control; RT, room temperature.


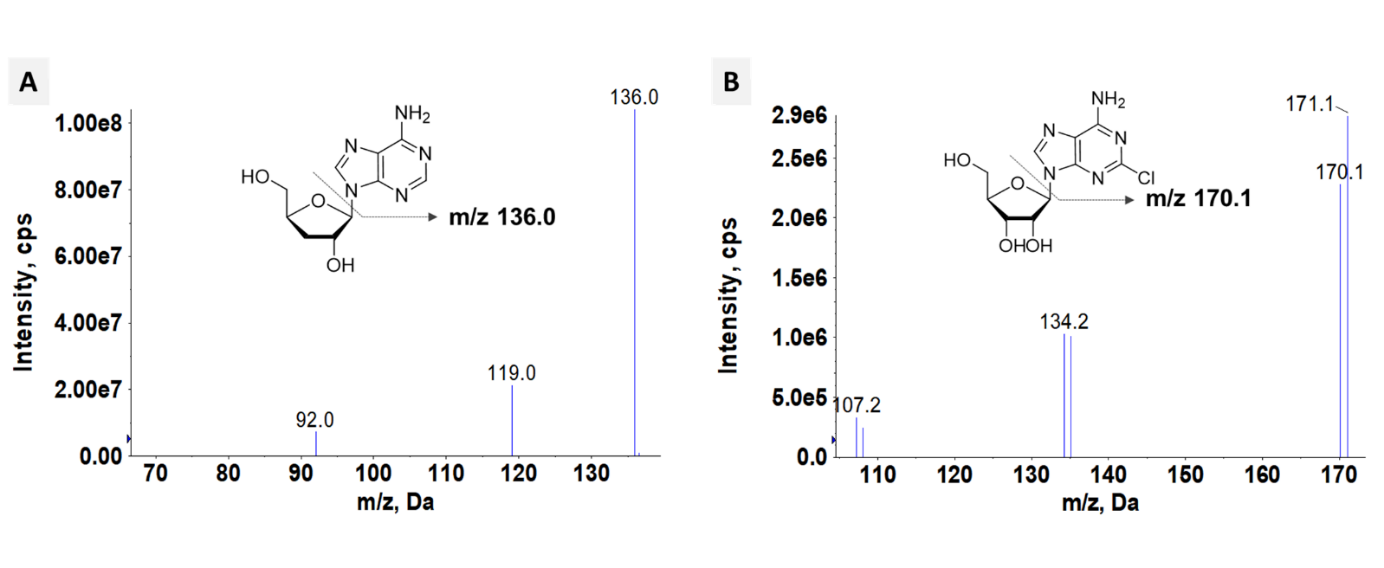


**Figure 1. Product ion mass spectra with proposed fragmentation**. **A**, protonated cordycepin (*m/z* = 252.2 🡪 136.0); **B**, protonated 2-chloroadenosine (*m/z* = 302.1 🡪 170.1).


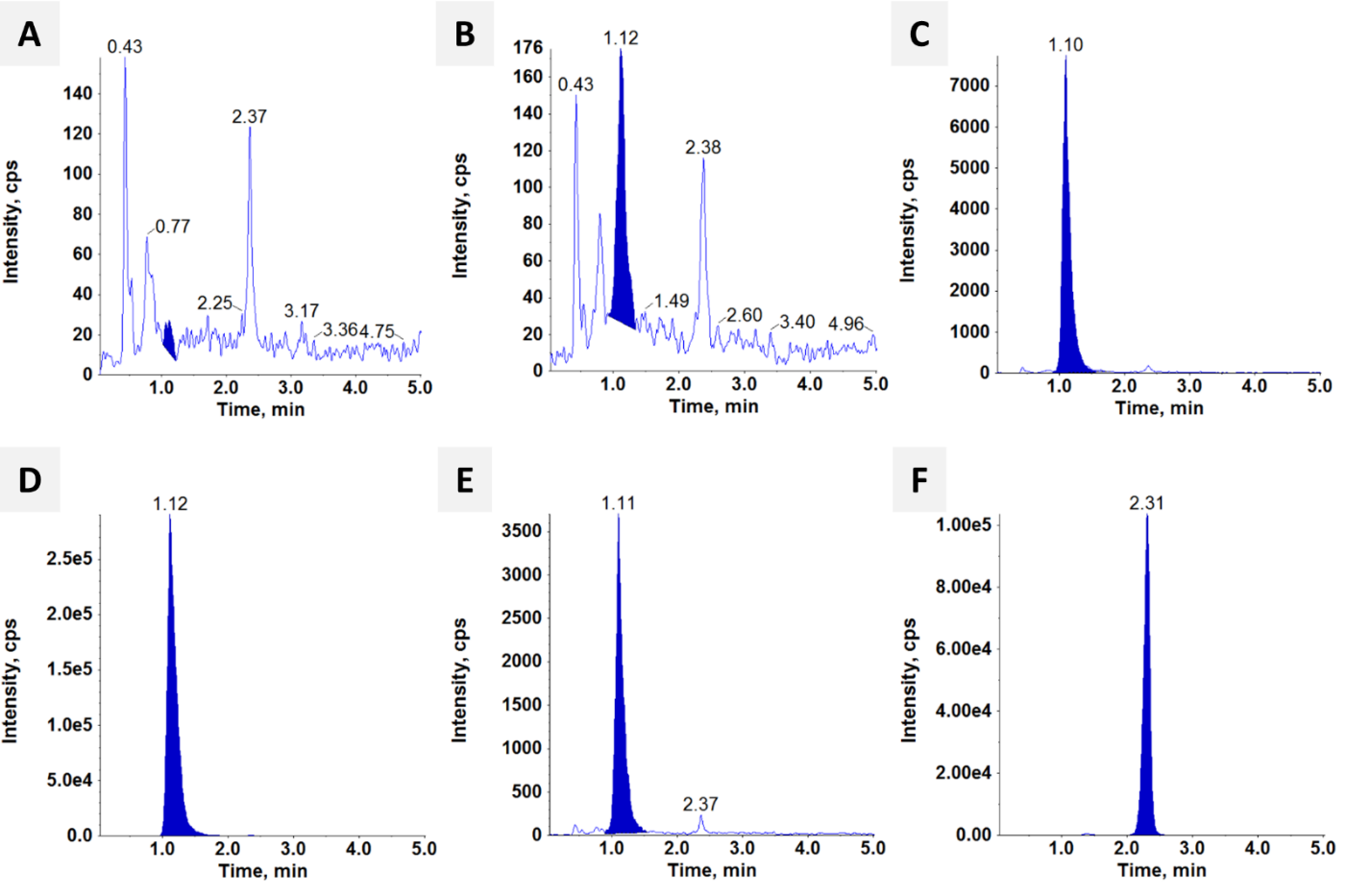


**Figure 2. Representative LC-MS/MS chromatograms from the final developed bioanalytical method. A**, blank rat plasma; **B**, rat plasma spiked with 2 ng/mL cordycepin (LLOQ); **C**, rat plasma spiked with 80 ng/mL cordycepin (MQC); **D**, rat plasma spike with 4000 ng/mL cordycepin (HQC); **E**, rat plasma sample at 15 min after intravenous injection of 20 mg/kg cordycepin; **F,** chromatogram for internal standard (2-chloroadenosine).

**Supplementary Information 4.** Representative chromatograms for bioanalytical methods used for 3’-deoxyinosine and CordyTP


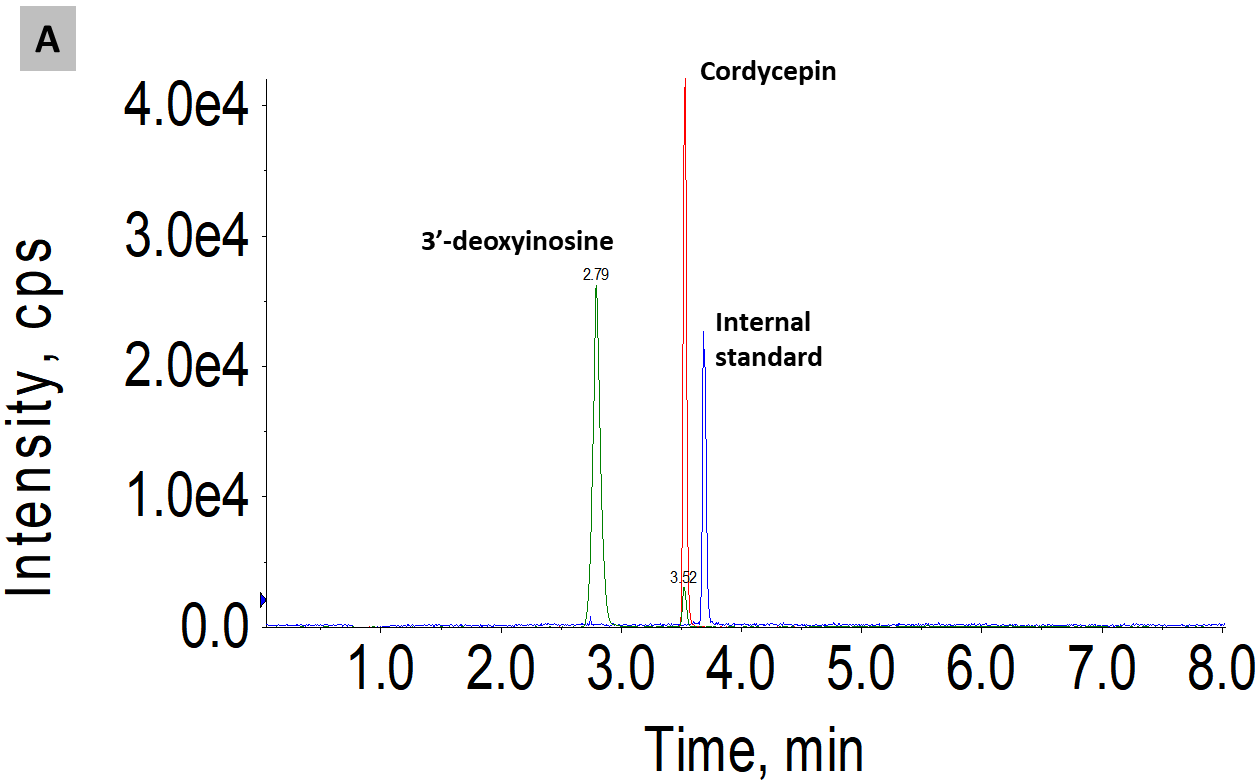


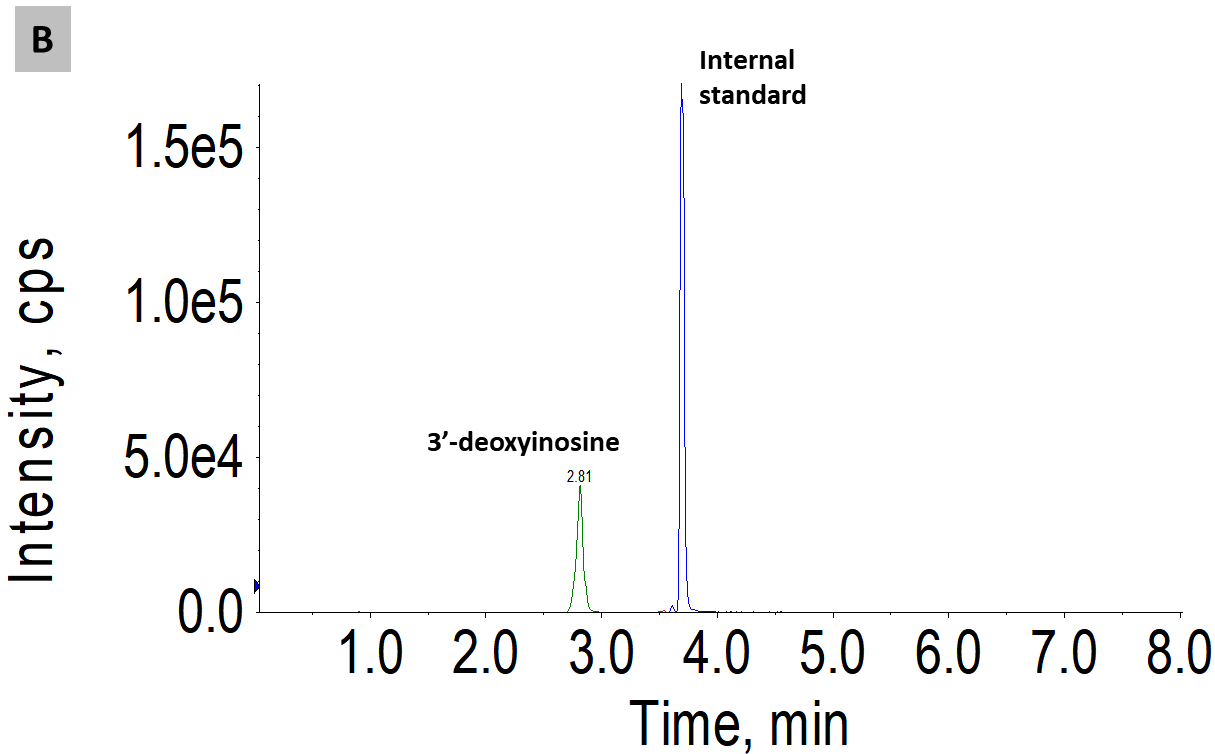


**Figure 1. Representative LC-MS/MS chromatograms of 3’-deoxyinosine, cordycepin and internal standard. A**, chromatogram from rat plasma obtained at 2 min following intravenous administration of cordycepin at 20 mg/kg; **B**, chromatogram from rat plasma obtained at 15 min following oral administration of cordycepin at 80 mg/kg.


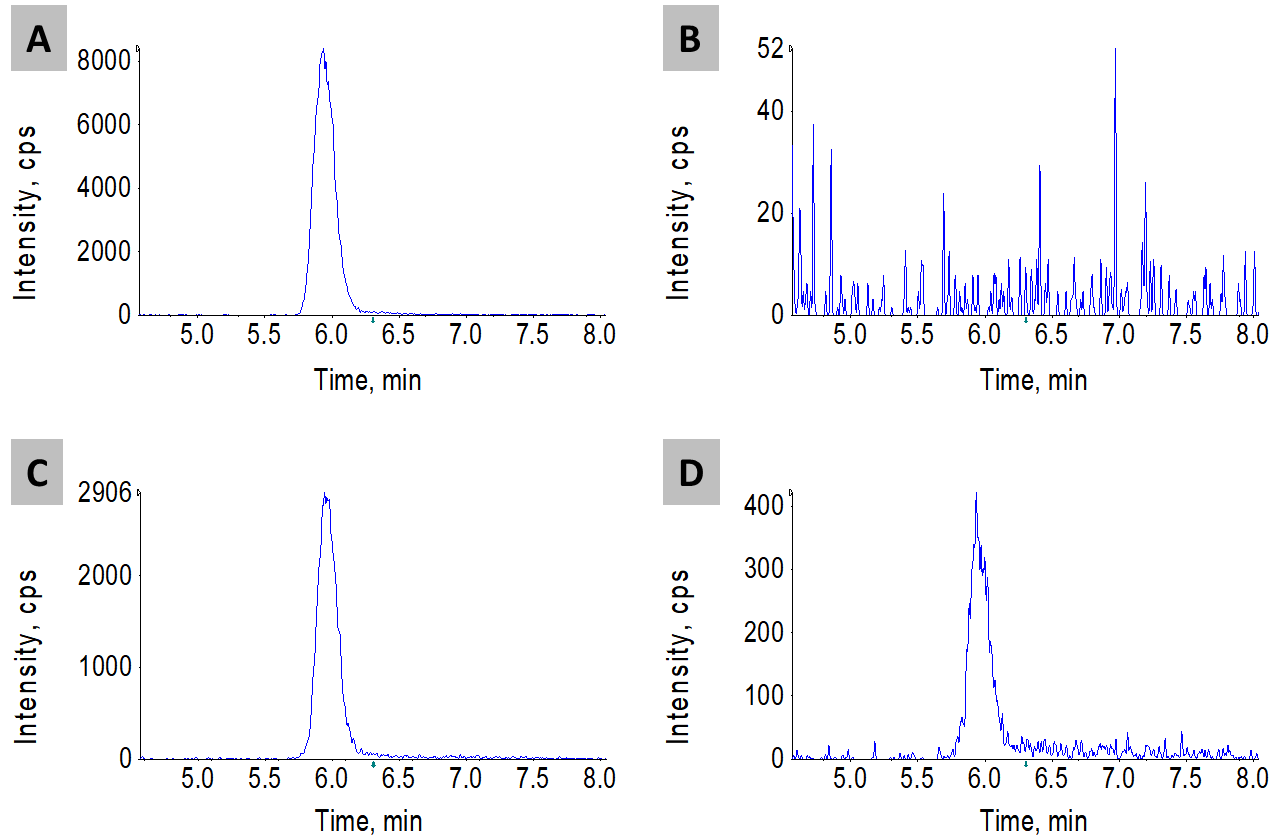


**Figure 2. Representative LC-MS/MS chromatograms of CordyTP obtained from RAW 264.7 cell lysates. A**, cell lysate spiked with CordyTP for calibration curve; **B**, blank cell lysate; **C**, cell lysate obtained following treatment with cordycepin; **D**, cell lysate obtained following treatment with 3’-deoxyinosine.

**Supplementary Information 5.** Equations for calculation of pharmacokinetic parameters

For calculation of pharmacokinetic parameters, the plasma concentration-time curve was plotted in a semi-log scale. From the plot, the elimination rate constant (*k_e_*) was calculated. Elimination half-life (*t_1/2_*) wasthen calculated using the following equation:

$$t_{1/2}=-\frac{\ln\left( 2 \right)}{k_{e}}=-\frac{0.693}{k_{e}}$$

Area under the plasma concentration-time curve (AUC) was calculated using the trapezoidal method with linear-up-log-down approach. In this approach, two separate equations were applied to one plasma pharmacokinetic profile: linear trapezoidal method was used when concentration was increasing over time; logarithmic trapezoidal method was used when concentration was decreasing over time. The equation for linear trapezoidal method was as follows:

$$AUC=\frac{1}{2}(C_{1}+C_{2})(t_{2}-t_{1})$$

where, *C_1_* and *C_2_* are plasma concentration levels at time *t_1_* and *t_2_* (*t_2_*>*t_1_*). The equation for logarithmic trapezoidal method was as follows:

$$AUC=\frac{C_{1}-C_{2}}{\ln\left( C_{1} \right)-\ln\left( C_{2} \right)}(t_{2}-t_{1})$$

Note that logarithmic trapezoidal method was used when concentration was decreasing over time, so the equation was used under assumption that *C_1_*>*C_2_*. AUC_0🡪t_ represents the AUC calculated from time zero until the last observed time point. The AUC from time zero to infinity (AUC_inf_) was calculated by the following equation:

$${AUC}_{inf}={AUC}_{0\to t}+\frac{C_{last}}{k_{e}}$$

where, C_last_ is the concentration at the last observed time point. Plasma clearance (CL) was calculated using the following equation:

$$CL=\frac{Dose}{AUC}$$

where, AUC_0🡪t_ was used for cordycepin and AUC_inf_ was used for 3’-deoxyinosine. Area under the first moment curve from time zero to time extrapolated to infinity (AUMC_inf_) was calculated by the following equation:

$${AUMC}_{inf}=\left[ \sum_{1}^{n} \frac{C_{i}+C_{i+1}}{2}\cdot(t_{i+1}-t_{i}) \right]+\frac{C_{last}\cdot t_{last}}{k_{e}}+\frac{C_{last}}{k_{e}^{2}}$$

where, *C_i_* is the plasma concentration at time *t_i_*, C_last_ is the plasma concentration at the last observed time point (t_last_). Volume of distribution at steady state (*V_ss_*) was calculated by the following equation:

$$V_{ss}=\frac{Dose\cdot{AUMC}_{inf}}{{AUC}_{inf}^{2}}$$
